# THE BOLD PUBLIC HEALTH CENTERS OF EXCELLENCE ON DEMENTIA: MOBILIZING PUBLIC HEALTH ACTION TO ADDRESS DEMENTIA

**DOI:** 10.1093/geroni/igac059.272

**Published:** 2022-12-20

**Authors:** Matthew Baumgart, Nora Super

## Abstract

Under the BOLD Infrastructure for Alzheimer’s Act, passed by Congress in 2018, the Centers for Disease Control and Prevention (CDC) established three Public Health Centers of Excellence (PHCOEs) to address Alzheimer’s disease and related dementias. Awarded in the Fall of 2020, the three PHCOEs are: (1) Dementia Caregiving; (2) Dementia Risk Reduction; and (3) Early Detection of Dementia. Each PHCOE is charged with identifying, translating, and disseminating research findings and evidence-based public health best practices in their particular area of focus – and then working with state, local, and tribal public health agencies across the country to undertake dementia-related activities in their communities. This Symposium will feature an overview by Dr. McGuire of the BOLD Act and CDC’s efforts to address Alzheimer’s and related dementia, followed by presentations on the work and the plans of each of the PHCOEs. Dr. Gaugler will discuss the Caregiving PHCOE’s efforts to support unpaid caregivers of those living with dementia, including how best to meet the caregiving needs of diverse communities. Mr. Baumgart will present the Risk Reduction PHCOE’s findings on the state of the evidence on risk reduction and how that evidence is being translated to public health action. Dr. Chodosh/Dr. Borson will review the plans of the Early Detection PHCOE to increase awareness of the value of earlier detection and the best ways of conducting detection strategies. Finally, Ms. Super will discuss how this public health work complements the work of the aging community in addressing Alzheimer’s and related dementias.

